# Electrical Activity in a Time-Delay Four-Variable Neuron Model under Electromagnetic Induction

**DOI:** 10.3389/fncom.2017.00105

**Published:** 2017-11-21

**Authors:** Keming Tang, Zuolei Wang, Xuerong Shi

**Affiliations:** ^1^School of Information Engineering, Yancheng Teachers University, Yancheng, China; ^2^School of Mathematics and Statistics, Yancheng Teachers University, Yancheng, China

**Keywords:** Hindmarsh–Rose neuron model, electromagnetic induction, magnetic flow, time-delay, multiple modes

## Abstract

To investigate the effect of electromagnetic induction on the electrical activity of neuron, the variable for magnetic flow is used to improve Hindmarsh–Rose neuron model. Simultaneously, due to the existence of time-delay when signals are propagated between neurons or even in one neuron, it is important to study the role of time-delay in regulating the electrical activity of the neuron. For this end, a four-variable neuron model is proposed to investigate the effects of electromagnetic induction and time-delay. Simulation results suggest that the proposed neuron model can show multiple modes of electrical activity, which is dependent on the time-delay and external forcing current. It means that suitable discharge mode can be obtained by selecting the time-delay or external forcing current, which could be helpful for further investigation of electromagnetic radiation on biological neuronal system.

## Introduction

As the important components of neuronal system, neurons have a pivotal role in regulating the dynamical behaviors of neuronal system. Therefore, the complex behaviors of neurons have been investigated extensively (Ozer and Ekmekci, 2005; Postnov et al., 2009; Volman et al., 2011, 2012; Buschman et al., 2012; Lv et al., 2016; Wang et al., 2017). For this reason, some neuron models have been established and analyzed theoretically (Hodgkin and Huxley, 1952; Morris and Lecar, 1981; Hindmarsh and Rose, 1982; Izhikevich, 2004; Herz et al., 2006; Ibarz et al., 2011), some of which are beneficial for understanding the mode transition in electric activities of the neuron. For example, Hodgkin–Huxley (HH) model (Hodgkin and Huxley, 1952) and Morris–Lecar (ML) model (Morris and Lecar, 1981) are used to depict the effect of ion channels on the membrane potential of neurons. A three variable Hindmarsh–Rose (HR) neuron model is obtained (Hindmarsh and Rose, 1982), which is simplified from HH neuron model and is used to reproduce the main properties of neuronal activities. It is also effective for bifurcation analysis (Pinto et al., 2000; Storace et al., 2008; Gu et al., 2014).

To our knowledge, as a mathematical neuron model, the three-variable HR neuron model could not describe the effect of ion channel. According to this, some researchers proposed four-variable HR neuron models (Moujahid et al., 2011; Rech, 2012), which can better describe the dynamical behaviors of neurons and can be verified by experimental results (Gu and Pan, 2015). Indeed, because the neuron in neuronal system is in a complex situation, the electrical activity of neurons is too complex and more factors should be considered. For example, according to the Faraday's law of induction, the change of action potentials in neurons can generate magnet field in the media, while the magnet field could affect the electrical activity of neurons as a result of feedback effect. It means that the distribution of electromagnetic field inner and external of neurons could be altered owing to the change of membrane potentials of neurons. Therefore, the magnetic flux across membrane should be considered when exploring the dynamical behaviors of neurons. To probe the effect of electromagnetic induction on membrane potential of neurons, Lv et al. put forward a four-variable neuron model by introducing a new variable considering the magnetic flux and discussed the electric activities of it (Lv et al., 2016).

It is often thought that neuronal system can be with good memory to keep normal activities, while time-delay is often used to describe the memory effect. As we all know, magnetic field or magnet flux storage may be associated with the memory effect. In fact, when signals are propagated between neurons even within one neuron, there often exists time-delay. But, until now, the effect of time-delay on the electric activities of neurons has been seldom explored. To better describe the behaviors of neurons, more possible factors should be considered in investigating the electric activities of neurons.

Based on above, a time-delay four-variable neuron model under magnetic flow is proposed and to be investigated via numerical simulations. Other parts of this paper are arranged as follows. In section Model Description, the neuron model to be discussed is given. In section Numerical Simulation Results and Discussions, the electric activities of the addressed neuron model are discussed with the change of external forcing current and time-delay under the effect of magnetic flow. Finally, conclusions are drawn in section Conclusions.

## Model description

Hindmarsh–Rose neuron model is a three-variable model (Hindmarsh and Rose, 1982), which can be described as

(1) $$\{\begin{array}{l} \dot{x}=y-ax^{3}+bx^{2}-z+I_{ext}\\ \dot{y}=c-dx^{2}-y\;,\\ \begin{array}{l} \dot{z}=r\left[S\left(x+k\right)-z\right] \end{array} \end{array}$$

where *x* is membrane potential, *y* is recovery variable for slow current and *z* is the adaption current. *I*_*ext*_ is the external forcing current. *a*, *b*, *c*, *d*, *r*, *S*, *k* are system parameters and *k* is used to adjust the resting state.

Based on HR neuron model, by introducing a variable for magnetic flow, a four-variable HR neuron model (Gu and Pan, 2015) is designed to describe the effect of electromagnetic induction on neuronal activities, which can be rewritten as

(2) $$\{\begin{array}{l} \dot{x}=y-ax^{3}+bx^{2}-z+I_{ext}-k_{1}\rho \left(w\right)x\\ \dot{y}=c-dx^{2}-y\;,\\ \begin{array}{l} \dot{z}=r\left[S\left(x+k\right)-z\right]\\ \dot{w}=k_{2}x-k_{3}w \end{array} \end{array}$$

where ρ(*w*) = (α + 3β*w*^2^) is the memory conductance of a magnetic flux-controlled memristor and is used to describe the coupling between magnetic flux as well as membrane potential of neurons. The fourth variable *w* can describe the magnetic flux across membrane. *k*_1_, *k*_2_, and *k*_3_ represents the interaction parameters between the cell potential and the magnetic flux. Some dynamical behaviors of the membrane potential in this model have been discussed (Gu and Pan, 2015). Model (2) could be helpful for further investigating the effect of electromagnetic radiation on biological tissue. On the other hand, HR system is a slow-fast system. When the slow oscillation of *z* drives the fast subsystem (*x, y*) through periods of oscillatory and quiescent behavior, there may be a time lag. Therefore, it is necessary to investigate the dynamical behaviors of the neuron model (2) with time-delay, which can be described as

(3) $$\{\begin{array}{l} \dot{x}=y-ax^{3}+bx^{2}-z(t-\tau )-k_{1}\rho \left(w\right)x+I_{ext}\\ \dot{y}=c-dx^{2}-y\;,\\ \begin{array}{l} \dot{z}=r\left[S\left(x+k\right)-z\right]\\ \dot{w}=k_{2}x-k_{3}w \end{array} \end{array}$$

where τ is the time-delay.

## Numerical simulation results and discussions

In this section, considering the external forcing current and time-delay, which play an important role in neural system, the dynamical behaviors of neuron system (3) is investigated with the change of external forcing current *I*_*ext*_ or the time-delay τ. Numerical simulations are given to analyze the relation between the dynamics and the external forcing current *I*_*ext*_ or the time-delay τ. In the simulations, the system parameters are taken as *a* = 1.0, *b* = 3.0, *c* = 1.0, *d* = 5.0, *r* = 0.006, *S* = 4.0, *k* = 1.6, *k*_1_ = 0.01, *k*_2_ = 1.0, *k*_3_ = 6.2, α = 0.4, β = 0.01 and the initial values of system (3) is chosen as (*x*(0), *y*(0), *z*(0), *w*(0)) = (0.5, 0.2, 0.8, 0.1). The time step is selected as *h* = 0.01 and the time series of membrane potential *x* are calculated via fourth-order Runge-Kutta for different parameters.

**Figure 1:**
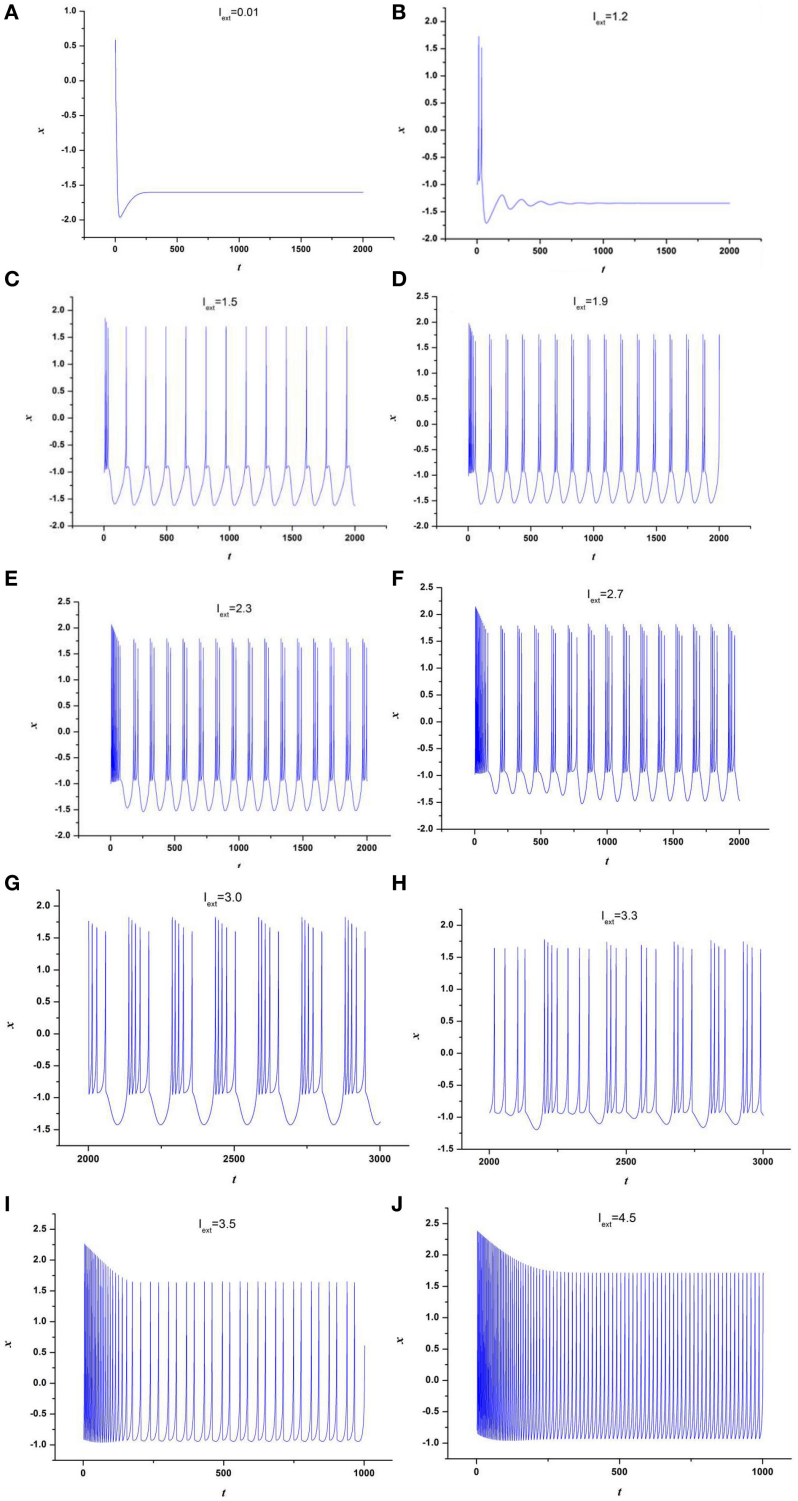
Time series of *x* in neuron system (3), **(A)** *I*_*ext*_ = 0.01, **(B)** *I*_*ext*_ = 1.2, **(C)** *I*_*ext*_ = 1.5, **(D)** *I*_*ext*_ = 1.9, **(E)** *I*_*ext*_ = 2.3, **(F)** *I*_*ext*_ = 2.7, **(G)** *I*_*ext*_ = 3.0, **(H)** *I*_*ext*_ = 3.3, **(I)** *I*_*ext*_ = 3.5, **(J)** *I*_*ext*_ = 4.5.

At first, the effect of external forcing current on the dynamical behaviors of neuron model (3) is investigated. For this end, the time series for membrane potential are calculated for different external forcing currents *I*_*ext*_ while the time-delay is taken as τ = 1, which are drawn in Figure 1. Figure 1 indicates that neuron system (3) possesses various discharge modes. For small value of external forcing current, for example *I*_*ext*_ = 0.01, 1.2, the neuron system (3) gradually tend to quiescent (see Figures 1A,B). But with the increasing of external forcing current, the electrical activities in neuron system (3) can show different modes, for example, when *I*_*ext*_ = 1.5, 1.9, 2.3, 2.7, 3.0, 3.3, the time series of system (3) appears period-1 bursting, period-2 bursting, period-3 bursting, period-4 bursting, period-5 bursting and chaotic bursting, respectively (see Figures 1C–H), while *I*_*ext*_ = 3.5, 4.5, neuron system (3) behaves spiking and period-1 bursting, respectively (see Figures 1I,J). From Figure 1, we can obtain that neuron system (3) shows various electrical activities with the change of external forcing current *I*_*ext*_, such as stable state, period bursting with different period, chaotic bursting, spiking.

**Figure 2:**
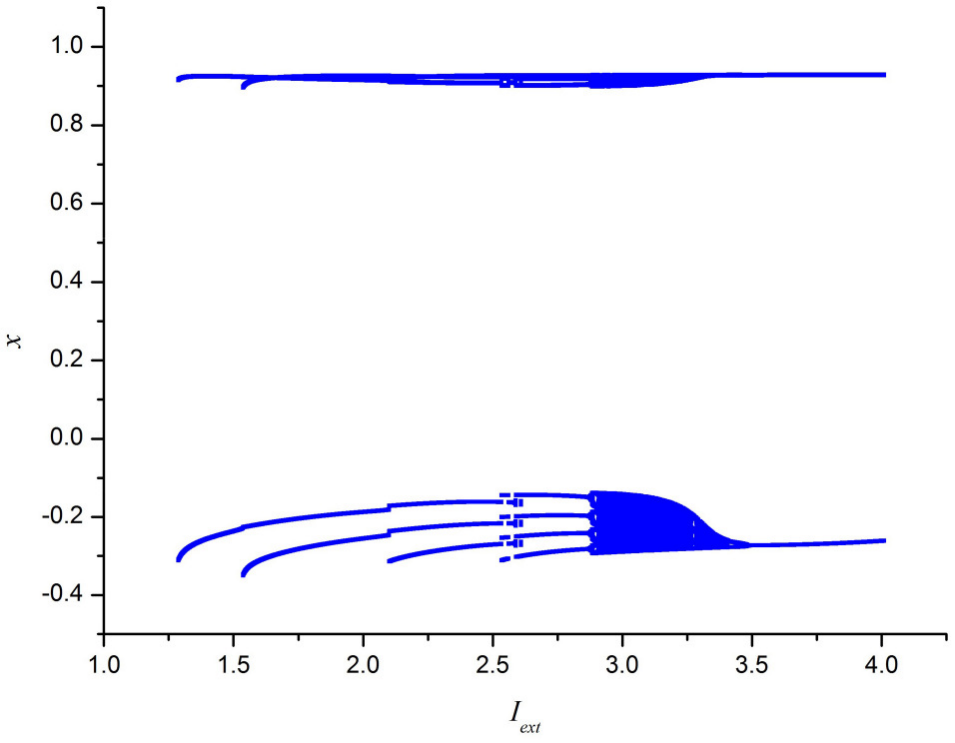
Bifurcation diagram of *x* (the Poncare section at *y* = 0) in neuron system (3) verses external forcing current *I*_*ext*_.

To further depict the effect of external forcing current on the electrical activities of membrane potential in neuron system (3), the external forcing current *I*_*ext*_ is taken as the bifurcation parameter and the bifurcation diagram of *x* verses *I*_*ext*_ is plotted in Figure 2. Figure 2 represents the Poncare section of membrane potential *x* at *y* = 0 with external forcing current *I*_*ext*_. From Figure 2, it is also known that the electrical activity in neuron system (3) varies with the change of external forcing current and shows diversity, which can also confirm that multiple modes in electrical activities of neuron system (3) can be arrived by selecting appropriate external forcing current.

**Figure 3:**
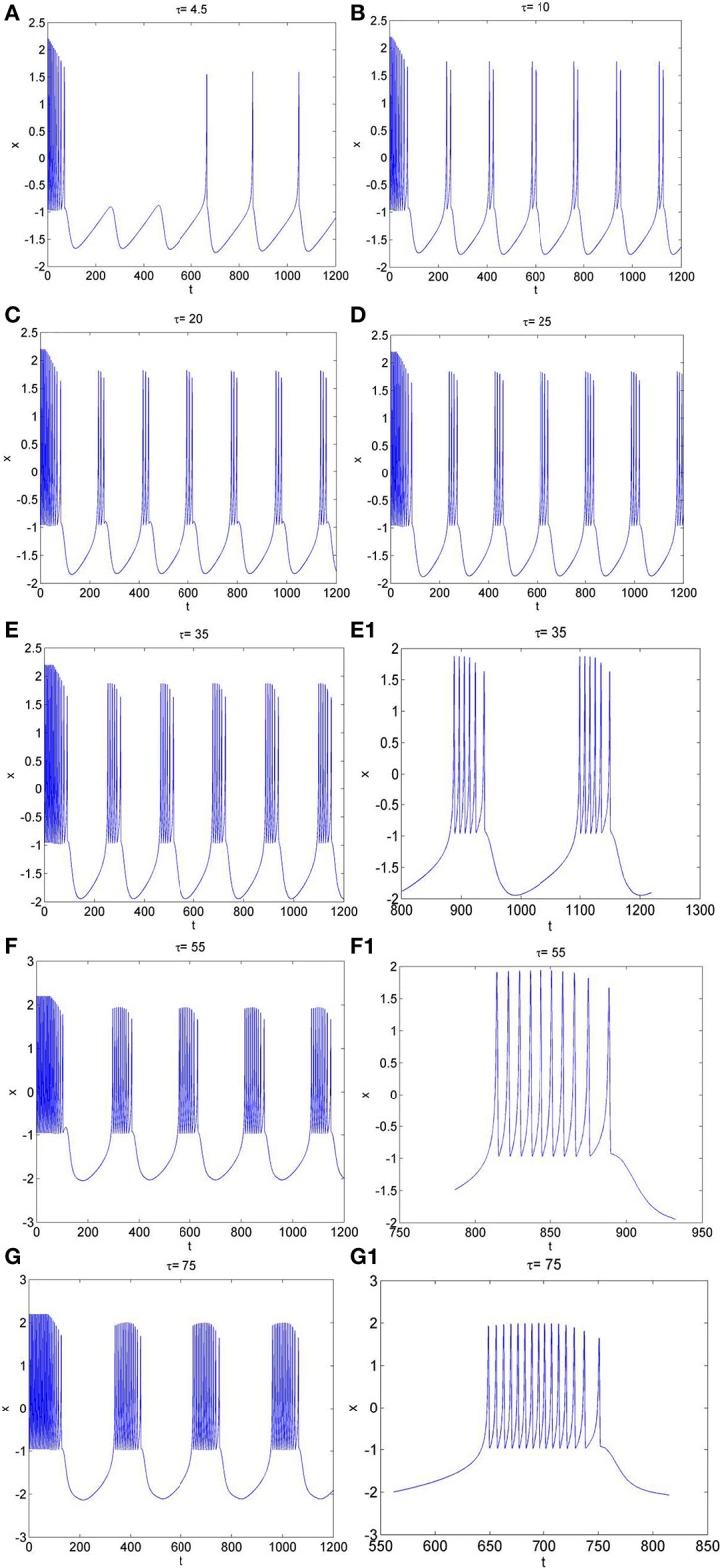
The time series of membrane potential *x* in neuron system (3) for *I*_*ext*_ = 1.2 and different values of time-delay, **(A)** τ = 4.5, **(B)** τ = 10, **(C)** τ = 20, **(D)** τ = 25, **(E)** τ = 35, **(E1)** Partial enlargement of **(E)**, **(F)** τ = 55, **(F1)** Partial enlargement of **(F)**, **(G)** τ = 75, **(G1)** Partial enlargement of **(G)**.

Secondly, the effect of time-delay on the dynamics of neuron system (3) is studied. The system parameters are taken as above. The external forcing current is fixed as *I*_*ext*_ = 1.2 and time-delay τ is chosen as different values. The time series of the membrane potential in neuron system (3) are calculated and given in Figure 3. From Figures 1B, 3, it can be obtained that, neuron system (3) tends to be stable when *I*_*ext*_ = 1.2 and time-delay τ = 1, but with the increasing of time-delayτ, even if the external forcing current *I*_*ext*_ keeps as a constant, the dynamical behaviors of membrane potential in neuron system (3) gradually shows multi-period bursting. Furthermore, Figures 3B–G demonstrate that, with the increase of τ, the time of quiescent between two adjacent bursting discharges becomes longer and longer, while the spiking frequency in one bursting discharge gets bigger and bigger.

**Figure 4:**
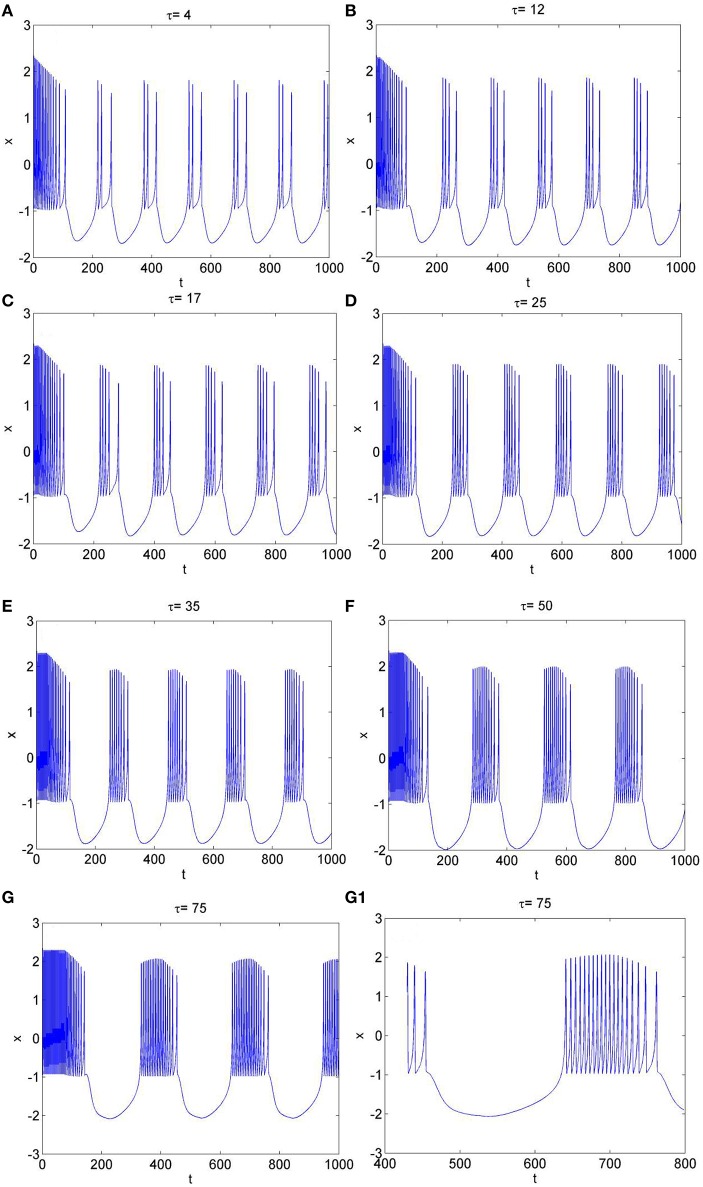
The time series of membrane potential *x* in system (3) for *I*_*ext*_ = 1.9 and different values of time-delay, **(A)** τ = 4, **(B)** τ = 12, **(C)** τ = 17, **(D)** τ = 25, **(E)** τ = 35, **(F)** τ = 50, **(G)** τ = 75, **(G1)** Partial enlargement of **(G)**.

**Figure 5:**
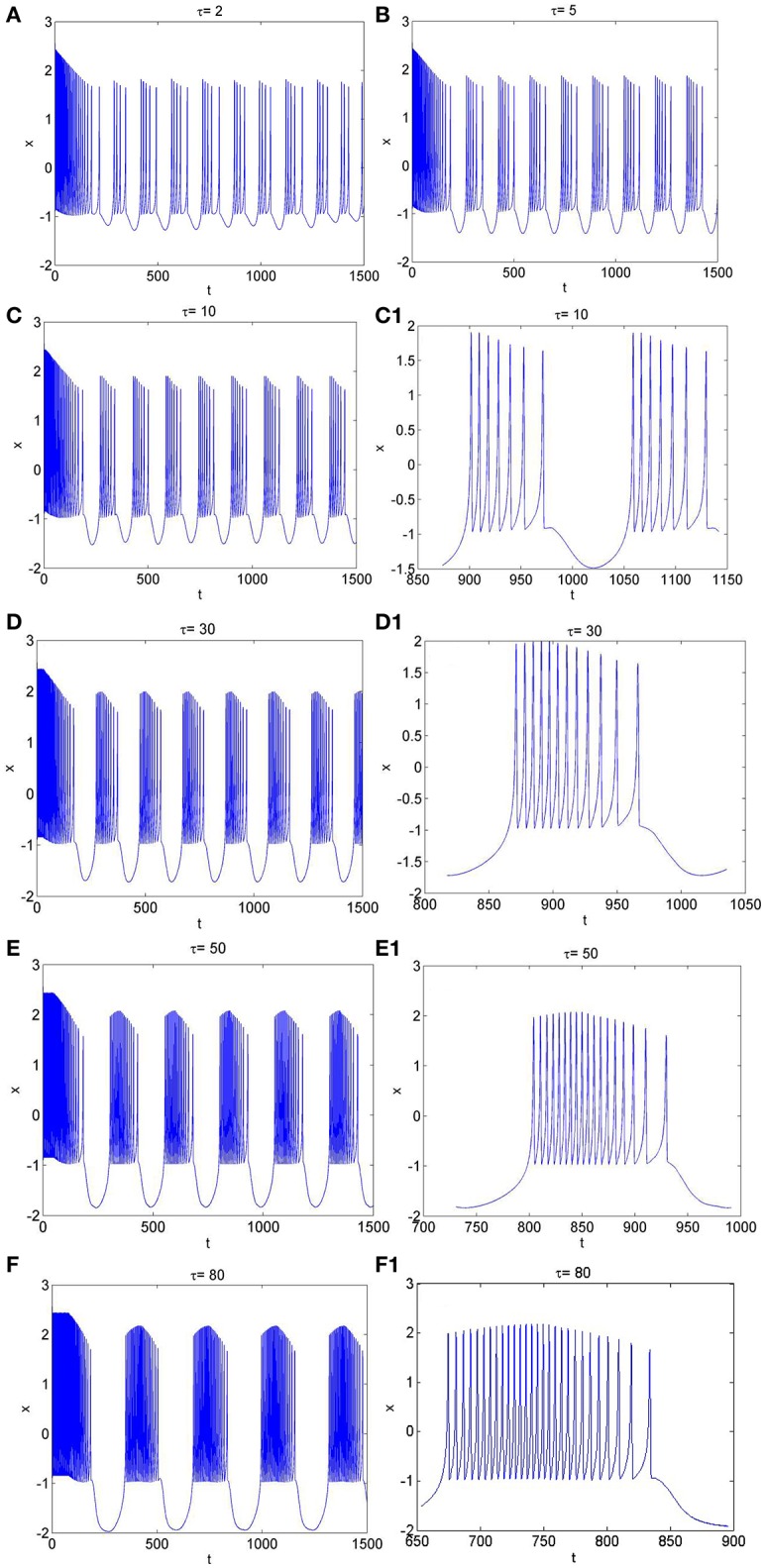
The time series of membrane potential in neuron system (3) for *I*_*ext*_ = 3.2 and different values of time-delay, **(A)** τ = 2, **(B)** τ = 5, **(C)** τ = 10, **(C1)** Partial enlargement of **(C)**, **(D)** τ = 30, **(D1)** Partial enlargement of **(D)**, **(E)** τ = 50, **(E1)** Partial enlargement of **(E)**, **(F)** τ = 80, **(F1)** Partial enlargement of **(F)**.

To further explore the rhythm of electrical activity, *I*_*ext*_ = 1.9 and *I*_*ext*_ = 3.2 are chosen respectively. The time series of membrane potential in neuron system (3) are calculated and depicted in Figures 4, 5, respectively. Figure 1D means that neuron system (3) shows dynamics of 2-period bursting when *I*_*ext*_ = 1.9 and time-delay τ = 1, while Figures 4A–G indicates that, if *I*_*ext*_ is fixed at 1.9, neuron system (3) can be provided with multi-period bursting for different values of time-delay τ, such as period-3 bursting for τ = 4, period-4 bursting for τ = 12, period-5 bursting for τ = 17, period-6 bursting for τ = 25, period-8 bursting for τ = 35, period-12 bursting for τ = 50, period-19 bursting for τ = 75. From these phenomena, it is obvious to see that, with increasing of time-delay τ, bursting period becomes longer and longer as well as the spiking frequency gets larger and larger. It means that, when neuron system (3) is in periodic state, by selecting the time-delay and external forcing current, certain bursting frequency can be obtained.

Similarly, from Figures 1H, 5A–F, it is known that, when neuron system (3) is in chaotic, the dynamical behaviors of it can also show diversity with the change of time-delay for fixed value of external forcing current, such as period-5 bursting for τ = 2, period-6 bursting for τ = 5, period-7 bursting for τ = 10, period-12 bursting for τ = 30, period-18 bursting for τ = 50, period-28 bursting for τ = 80. It means that the larger the time-delay is, the larger the bursting period of the electrical activity in neuron system (3) becomes and the larger the spiking frequency in one bursting is.

## Conclusions

In this paper, the electrical activity of a time-delay 4-D neuron system under electromagnetic induction is proposed and investigated via numerical simulations. The effect of the time-delay and external forcing current on the dynamical behaviors of the addressed neuron system is discussed. Main conclusions are given as following aspects.

1. A time-delay 4-D neuron system under electromagnetic induction is addressed by using magnetic flux.
2. When time-delay is τ = 1, the electrical activity of the proposed neuron system (3) is dependent on the external forcing current. With the change of external forcing current, the neuron system (3) shows multiple discharge modes, such as stable state, period bursting, chaotic bursting.
3. When external forcing current is fixed, the change of time-delay has greater impact on the electrical activity of neuron system (3). Whether neuron system (3) is in period bursting or chaotic bursting, by choosing different values of time-delay, neuron system (3) can be provided with multi-period bursting.
4. By selecting appropriate time-delay and external forcing current, neuron system (3) can give appropriate dynamical response with different modes.

The results suggest that the addressed neuron model can expand the parameter region to generate complex modes of electrical activity. Furthermore, the network of this model could be used to investigate the collective behaviors of neurons of brain and central nervous system, and the potential mechanism for disease induced by electromagnetic radiation could be explained.

## Author contributions

KT carried out the numerical simulations. ZW analyzed the experimental results and wrote the manuscript. XS polished the language of the paper.

### Conflict of interest statement

The authors declare that the research was conducted in the absence of any commercial or financial relationships that could be construed as a potential conflict of interest.
